# Hemispheric Asymmetries in Biodiversity—A Serious Matter for Ecology

**DOI:** 10.1371/journal.pbio.0020406

**Published:** 2004-11-16

**Authors:** Steven L Chown, Brent J Sinclair, Hans P Leinaas, Kevin J Gaston

## Abstract

Although the poles are less diverse than the tropics, this decline shows substantial asymmetries between the hemispheres, suggesting that responses to environmental change may differ substantially in the north and the south.

Penguins have been receiving a lot of bad press lately. They are considered somehow counter, spare, strange. Unlike most plant and animal groups, they do not show a peak of species richness towards the equator and a decline towards the poles. This more conventional spatial pattern is conveniently known as the latitudinal diversity gradient because of the strong covariance of richness and other measures of biodiversity that it describes. It is one of the most venerable, well-documented, and controversial large-scale patterns in macroecology (Willig et al. 2003). Equatorial peaks in species richness have characterised the planet since the Devonian (408–362 million years ago) (Crame 2001) and are typical of a wide range of both terrestrial and marine plants and animals (Gaston 1996; Willig et al. 2003). Despite the fact that this pattern has been documented since the late 1700s, sustained interest in both the regularity of the pattern and its likely underlying mechanisms is relatively modern. The realisation that human activity is posing substantial threats to biodiversity has quickened the pace of this interest (Willig et al. 2003). Where the peaks in richness lie (biodiversity hotspots), how these peaks relate to centres of endemism (areas that support large numbers of species that occur nowhere else), and how these patterns are likely to change through time, especially in the face of major environmental change, are major concerns. Without such knowledge, conservation is unlikely to succeed.

Although spatial patterns in biodiversity, and particularly the latitudinal gradient, are increasingly well documented for a range of taxa, the proposed mechanisms underlying these gradients remain controversial. In essence, the multitude of mechanisms proposed to explain diversity gradients can be reduced to three categories: historical, ecological, or null. Most significant in raising the temperature of recent discussions is the question of the relative importance of each of these major categories. Historical mechanisms are those that suggest that earth history (e.g., the opening of the Drake Passage and the cooling of Antarctica) and phylogenetic history have played major roles in generating current patterns in diversity, and tend to emphasise regional (and especially longitudinal) differences therein (Qian and Ricklefs 2004; Ricklefs 2004). Explanations involving ecological mechanisms often downplay the significance of such regional differences and give most attention to covariation between current diversity and variables such as energy and water availability, and to the ultimate mechanisms underlying this covariation (Hawkins et al. 2003; Currie and Francis 2004). By contrast, null models, and specifically the geometric constraints model, argue that the expected pattern of latitudinal variation in richness is not a uniform one, but rather a mid-domain peak, which is almost inevitably the outcome of the random placement of a set of variable species ranges within a bounded domain (Colwell et al. 2004; but see also Zapata et al. 2003). It is deviation from the mid-domain expectation that is then argued to be of most interest. In many cases the historical and ecological mechanisms might be difficult to disentangle, such as the historical effects of the establishment of the Antarctic Circumpolar Current, and its consequences for energy availability in the region today (Clarke 2003).

Nonetheless, juxtaposing these three major mechanisms raises several questions that could substantially inform the debate in many ways, but have enjoyed far less attention than debating the relative merits of each of them. The geometric constraints model suggests that, to the extent that there is symmetry in the continuity of land (or water) about the equator, declines in richness from the tropical peak should also be symmetrical, with any asymmetries in the latter matching those in the former. Indeed, most texts and reviews dealing with latitudinal diversity gradients only briefly mention hemisphere-related differences and focus instead on the general decline of diversity away from the tropics in both directions (e.g., Brown and Lomolino 1998; Willig et al. 2003). However, that diversity gradients in the two hemispheres might in many cases be highly asymmetric has long been appreciated (Gaston 1996). Although several historical hypotheses suggest reasons why this asymmetry should exist (reviewed in Brown and Lomolino 1998), differences in present ecological factors, such as temperature gradients and rainfall variation, might also explain such asymmetry. If ecological factors are important, then these asymmetries should show up not only in diversity patterns, but also at other levels in the ecological and genealogical hierarchies. From the perspective of ecological explanations for such spatial variation, the questions, then, are how common and strong are such asymmetries, how common are they in patterns of diversity, and what, if any, might be the ecological, rather than null or historical, mechanisms responsible for them?

## Continents and Climates

**Figure 1:**
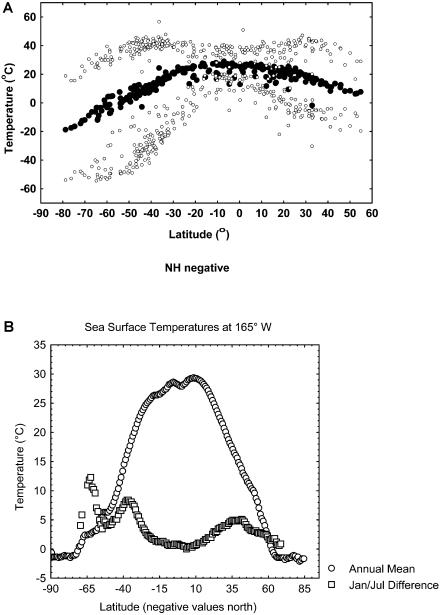
Temperature Variation with Latitude (A) Mean and absolute minimum and maximum temperatures across the New World.(B) Mean and absolute range in sea surface temperatures across the Pacific at 165° W.

The last 100 million years have seen both a substantial steepening in latitudinal diversity gradients and the fragmentation of continental land masses (Crame 2001). By 15 million years ago the continents had largely assumed their current positions and a latitudinal temperature gradient very similar to the present one had been established. Today, 70% of all land is in the northern hemisphere, and between latitudes 30° and 60° north, the ratio of water to land is about 1:1, whereas between 30° and 60° south, it is approximately 16:1. The continentality of the north and oceanicity of the south have considerable effects on the climates of the hemispheres, as has long been appreciated (Bonan 2002). Although there is obviously much local and mesoscale variation, terrestrial temperatures in the south (excluding Antarctica) are usually warmer, and much less extreme in terms of their absolute range, than those in the north (Figure 1A), especially over the winter months. Southern sites between 30° and 60° typically have mean July temperatures between 0 and 10 °C, whereas at similar latitudes in the north, mean January temperatures vary from −40 to 0 °C. In winter the smaller range of variation in the south is around a physically and biologically significant threshold: the freezing point of water. In the north, winter temperatures are more variable, but generally well below this point. Ocean water temperatures are much less variable than those on land, although variability in the ocean surrounding Antarctica is much reduced compared with that of the Arctic (Figure 1B).

Mean annual precipitation is spatially more complex. Overall, precipitation is slightly higher in the south than in the north, at least below 60° latitude. However, much of this precipitation falls over the ocean in the south, leaving the more temperate parts of the southern continents as dry as their northern counterparts (Bonan 2002). Spatial patterns in rainfall variability are also complex, but variability tends to be higher and predictability lower in southern areas. From a biological perspective the significant factor is not necessarily just the magnitude of the variance, but also the mean about which it occurs (Guernier et al. 2004).

Clearly, the spatial complexity of climatic variation is much greater than the present overview would suggest. However, these broad brush strokes capture the hemisphere-related variation that might be most significant from a biological perspective.

## Ecological Consequences

If differences in climates do cascade upwards to influence individuals, species, and broader scale patterns in diversity, their influence should be readily detectable at the level of species' life histories and distributions. In birds, large-scale geographic variation in life history variables, such as the incidence of cooperative breeding, extent of parental care, survival, and the timing of reproduction, has been studied for at least the past 50 years, and the mechanisms underlying this variation have been much debated. Taking phylogeny and the idiosyncrasies of the Australian avifauna into account, southern species typically lay small clutches and have long fledging periods, and it is often difficult to predict their date of first laying or, indeed, whether they will lay in a particular year at all (Covas et al. 1999; Russell et al. 2004). By contrast, northern species lay larger clutches and have shorter fledging periods, and laying date is more readily predicted, making investigations of phenological shifts associated with modern climatic change more straightforward (e.g., Crick et al. 1997).

These kinds of differences extend to other taxa. Thus, although the variation of metabolic rate with latitude is becoming increasingly well known for a variety of groups, Lovegrove (2000) has recently suggested, based on comparative work taking both species body mass and phylogeny into account, that unpredictability of resources associated with considerable inter-annual unpredictability in rainfall (in turn partly a consequence of El Niño–associated variability) has been responsible for the evolution of generally low metabolic rates in terrestrial mammals of most of the southern continents. Although El Niño effects are by no means restricted to these regions, it is perhaps low resource availability to start off with, associated with considerable unpredictability, that is of most significance (see also Guernier et al. 2004).

**Figure 2:**
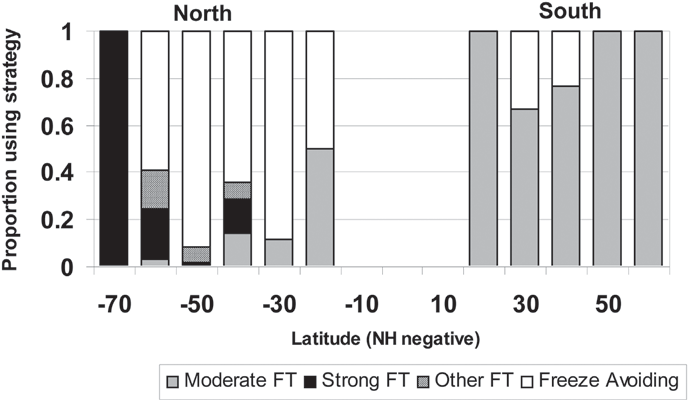
Latitudinal Variation in Cold Tolerance Strategies in Insects The proportion of insects, as a function of latitude, that are moderately freeze tolerant down to relatively high sub-zero temperatures (moderate FT), that are freeze tolerant down to low sub-zero temperatures (strong FT), that are freeze tolerant but that cannot be classified (other FT), and that are freeze avoiding.

Insect life histories also show hemisphere-related variation. Low-temperature-related diapause is virtually absent in southern species (e.g., Convey 1996), and metabolic rate–temperature relationships are much shallower in the south than the north (Addo-Bediako et al. 2002). The latter is a consequence of relatively cool growing seasons and lack of pronounced seasonality in the south. However, the clearest example of a hemispheric asymmetry is that of cold hardiness strategies (Sinclair et al. 2003). Insects can survive sub-zero temperatures either by tolerating internal ice formation or by reducing their freezing points to avoid ice formation altogether. Although there is further variation within each of these strategies, in general, freeze-avoiding species need to undergo substantial preparation for winter cold and consequently can take some time to emerge from the cold hardy state. This also seems to be true of strongly freeze-tolerant species that can survive freezing far below the point at which they actually freeze. By contrast, moderately freeze-tolerant species—those that can survive only a few degrees of freezing—appear to need little preparation for a freezing event and seem perfectly prepared to continue with their routine activities immediately after thawing. In northern cold climate areas, with the exception of the Arctic, where extremely low temperatures constrain insects to being strongly freeze tolerant, most cold hardy species avoid freezing, whereas in the south most are moderately freeze tolerant (Figure 2). Microclimates reveal why this is the case. As might be expected from macroclimatic variation, southern temperate insects are faced with regular freeze–thaw cycles (i.e., variation about 0 °C), including pronounced summer cold snaps, whereas the continental climates of many areas in the north mean that once temperatures decline below freezing for winter, they stay below this threshold.

**Figure 3:**
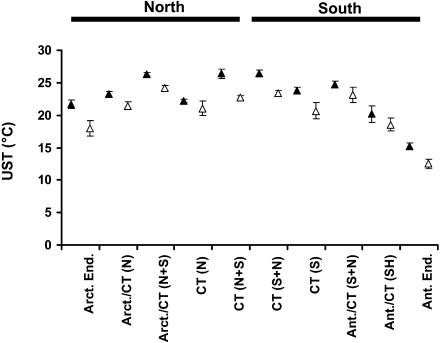
Variation in Upper Survival Temperatures of Macroalgae from across the Planet Mean and standard error of upper survival temperatures of macroalgae (open symbols, macrothalli; closed symbols, microthalli) from cold areas across the planet. Ant., Antarctic; Arct., Arctic; CT, cool temperate; End., endemic; N, northern hemisphere only; S, southern hemisphere only; N+S, occurrence in both hemispheres. Redrawn from Wiencke et al. (1994).

North–south asymmetries also show up in snowlines, treelines, the frost tolerance of trees, and the proportion of winter deciduous species (Woodward 1987; Körner 1998; Körner and Paulsen 2004). Indeed, such differences have long been appreciated for vegetation. In marine systems, one of the best-known asymmetries is the low upper thermal limit to performance and survival in Antarctic compared with Arctic ectotherms. This difference in limits to survival and performance is characteristic of fish, invertebrates, and macroalgae (e.g., Wiencke et al. 1994) (Figure 3). Asymmetries are also apparent in the geographic ranges of a wide variety of animals and plants. Rapoport's rule proposes that species ranges will be larger at high than at low latitudes (Stevens 1989). The pattern is thought to be a consequence of considerably greater temporal climatic (and especially temperature) variation at high latitudes, and the resulting need for broader physiological tolerances of individuals. These broad tolerances enable the species to which these individuals belong to occur across a wider range of sites than species at lower latitudes. However, if there is much less temporal temperature variation in the south than in the north, evidence for the rule should be less forthcoming in the southern hemisphere. This is indeed the case. Consistent increases in latitudinal extents with latitude are uncommon in the south, and Rapoport's rule is now largely considered to be a northern phenomenon (Gaston et al. 1998).

## Large-Scale Asymmetries in Biodiversity

In the years since Platnick (1992) suggested that the world is pear-shaped from a biodiversity perspective, with more rapid declines in richness from the equator in the northern than in the southern hemisphere, evidence that there are large-scale asymmetries in the latitudinal diversity gradient has been accumulating. Seed plant and mammalian family richness per unit area declines more steeply in the northern hemisphere than in the south (Woodward 1987; Gaston et al. 1995), and similar asymmetries, mostly at the species level, have been noted for other groups such as New World birds, several groups of insects, spiders, foraminiferans, and a variety of benthic marine taxa (Platnick 1992; Rex et al. 1993; Eggleton 1994; Blackburn and Gaston 1996; Culver and Buzas 2000; Rodriguero and Gorla 2004). Nonetheless, not all groups show these trends, and a recent meta-analysis, albeit one on a relatively coarse scale, failed to find consistent north–south differences in latitudinal gradients (Hillebrand 2004). Recent reviews, particularly of marine diversity, have pointed out the difficulty of making comparisons of this kind owing to sampling constraints (Clarke and Johnston 2003). However, it remains remarkable that even simple exercises—such as plotting richness values for different latitudes or latitudinal bands against each other for the hemispheres and examining the resulting relationship, or overlaying them on the same range of latitudes—rarely appear in the literature. Thus, it is not yet clear how common or strong hemisphere-related asymmetry is.

**Figure 4:**
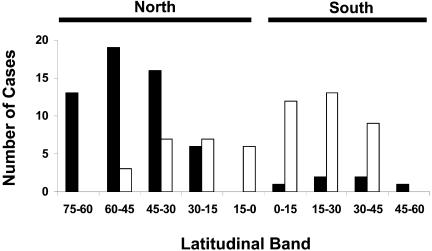
Latitudinal Variation in the Energy–Water Correlates for Species Richness Latitudinal distribution of energy–water correlates for species richness in which spatial variation in pure energy variables (closed bars), typically measured as temperature or potential evapotranspiration, or spatial variation in pure water availability variables (open bars), typically measured as rainfall or precipitation, best explains richness variation through space. Redrawn from Hawkins et al. (2003).

By contrast, it appears that proximate ecological correlates of diversity gradients differ considerably between north and south. Although both historical and ecological factors have led to variation in the numbers and identity of species across the globe (Ricklefs 2004), climate, and particularly energy and water availability, is a strong predictor of broad-scale patterns in species richness for both plants and animals. However, the extent to which energy and water availability constrain species richness varies. In a recent comparative analysis, Hawkins et al. (2003) showed that water availability is the key limiting component of richness for the southern hemisphere, but for temperate regions of the north, energy availability is more important (Figure 4). They ascribe this difference to the warmer and less thermally variable conditions of the southern hemisphere, which, as we have already noted, have considerable effects on species life histories.

**Figure 5:**
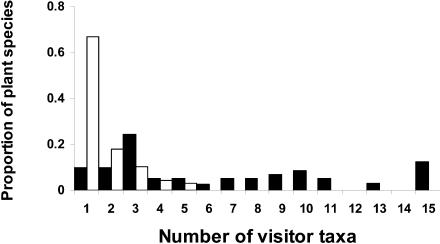
Number of Insect Species Pollinating Orchid Species in the Northern and Southern Hemispheres Europe and North America, closed bars, *n* = 41; southern Africa, open bars, *n* = 73. Redrawn from Johnson and Steiner (2003).

Of course, biodiversity is not just species richness, but also encompasses the ecological complexes of which species are a part. Although potential north–south asymmetries in interactions have not been widely explored, recent work is providing tantalising glimpses of such variation. Thus, it appears that on the basis of a straightforward (not phylogenetically corrected) comparative analysis, specialisation in plant–pollinator relationships is much greater in the south than in the north. European and North American orchids are typically visited by five species of insects, whereas in southern Africa the median is a single pollinator species per species of orchid (Johnson and Steiner 2003) (Figure 5). Insect–plant interactions might also vary in other ways between the hemispheres, as the rarity of showy autumn colours and the paucity of aphid species—which are thought by some to be a driver of these displays (Archetti and Brown 2004)—in south temperate areas suggests. Asymmetries in patterns of human disease point to similar hemisphere-related variation in interactions between organisms (Guernier et al. 2004).

## A World in Flux

**Figure 6:**
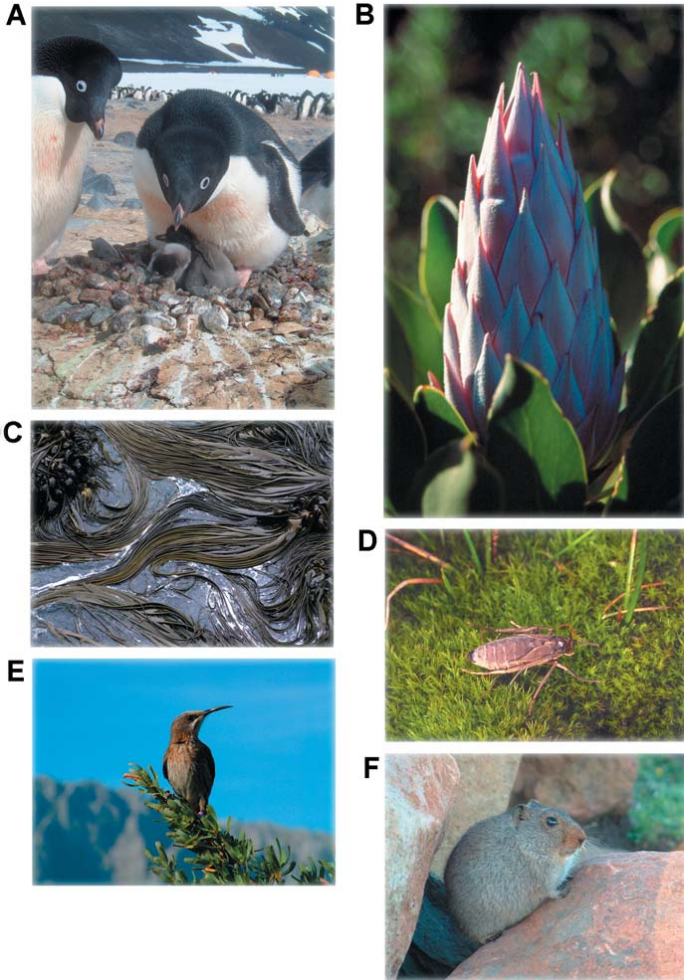
Biological Diversity in the Northern and Southern Hemispheres Regular differences between the northern and southern hemispheres in patterns of diversity show up in various groups such as the birds (A) (Adelie Penguin, Pygoscelis adeliae) and seed plant families (B) (King Protea, Protea cynaroides). North–south differences in life histories are also apparent in a diverse array of groups ranging from seaweeds (C) (Bull Kelp, Durvillaea antarctica) and insects (D) (the sub-Antarctic, flightless tineid moth Pringleophaga marioni) to birds (E) (Cape Sugarbird, Promerops cafer) and mammals (F) (Sloggett's Rat, Otomoys sloggetti, from the high Drakensberg in South Africa). (Photos: [A, C, and F] Brent J. Sinclair; [B and D] Steven L. Chown; [E] Mhairi L. McFarlane)

Despite considerable spatial complexity, there do seem to be regular north–south differences in species life histories and patterns of range size variation that are consistent with disparities in the climates of the two hemispheres (Figure 6). These differences extend to the proximate ecological mechanisms underlying spatial variation in species richness, and, in some cases, apparently to ecological interactions. However, what is less clear is the regularity and strength of north–south differences in spatial diversity patterns, and especially the latitudinal gradient in diversity, as well as the ways in which abiotic variation between the hemispheres might extend through the genealogical and ecological hierarchies to effect such differences. Indeed, if the extent to which abiotic differences between the hemispheres influence biodiversity patterns is to be better comprehended, several key issues deserve attention.

First, both phylogenetically independent and non-independent comparisons of life history traits and physiological variables across a variety of groups are required. Contrasting these approaches will provide considerable insight into how much of the signal is based on phylogenetic patterns, and how much on current ecological responses. Whilst in some cases data may be obtained from the literature, it is likely that new work will have to be undertaken, especially in the southern hemisphere, where the number of past investigations of such traits is generally much lower than in the north. Moreover, replicated studies using similar methods might substantially improve the signal-to-noise ratio, which can be weakened in “macrophysiological” or large-scale life history and physiological comparisons by the fact that different methods often lead to different outcomes.

Second, there is much to be said for the application of similar methods to investigations of large-scale, hemisphere-related patterns of species interactions. Differences like those in plant–pollinator systems discussed here might extend to other interactions in marine and terrestrial systems. Contrasting phylogenetically independent and non-independent comparisons are likely to provide much insight into the reasons for those asymmetries that are found.

Finally, comparisons of latitudinal gradients and their underlying correlates in the two hemispheres for the same taxon, sampled using similar methods, and investigated with methods that take cognisance of likely confounding effects are required. This approach will provide a means of determining whether asymmetries in the climates of the two hemispheres really do translate into differences in biodiversity patterns. Such an approach goes to the heart of the question of the processes underlying the latitudinal gradient in species richness, and could go a considerable way to teasing apart the importance of historical, ecological, and null explanations, and identifying the mechanisms that underlie them.

In our view, clarifying these issues is of considerable importance. What is at stake is not a set of arcane ecological questions, but rather questions that are central to determining whether ecological and conservation lessons learnt in one area can be applied more broadly. For example, it has been suggested that climate change will cause substantial extinctions in the near future (Thomas et al. 2004). Indeed, responses by species to such change, via phenological shifts and northward movement of species range margins, are well documented for northern hemisphere species (Parmesan and Yohe 2003). However, if there are substantial differences in abiotic environments such that patterns in diversity and their responses to change differ between hemispheres, then such shifts may not be of similar consequence in the south. To date, southern hemisphere studies represent less than 1% of the total in this field (Root et al. 2003), suggesting that it is not at all clear how the considerable biodiversity in the south will respond to future change. We find such a situation extraordinary. Thus, whilst penguins might at first appear counter, spare, and strange, they serve as a reminder that differences between the north and south might not be so much strange, as remarkable and worthy of closer attention.
